# Supplementary material for: Evaluation of convalescent plasma versus standard of care for the treatment of COVID-19 in hospitalized patients: study protocol for a phase 2 randomized, open-label, controlled, multicenter trial
Source: Trials. 2021 Jan 20;22:70. doi: 10.1186/s13063-020-05011-9 (PMC7816149; doi:10.1186/s13063-020-05011-9)
Supplement: Supplementary file 1 — Additional file 1. Clinical Trial sites and Transfusion centers. [file 13063_2020_5011_MOESM1_ESM.docx]

**Appendix 1:**

**Clinical Trial sites and Transfusion centers**

| **CLINICAL SITES** | | |
| --- | --- | --- |
| **#** | **name** | **CITY** |
| **1** | **H. U. Puerta de Hierro Majadahonda** | **Madrid** |
| **2** | **H. U.12 de Octubre** | **Madrid** |
| **3** | **H. u. Ramón y Cajal** | **Madrid** |
| **4** | **H. U. Clínico San Carlos** | **Madrid** |
| **5** | **H. C. U. Lozano Blesa Zaragoza** | **Zaragoza** |
| **6** | **H. U. Severo Ochoa** | **Madrid** |
| **7** | **H. U. Príncipe de Asturias** | **Madrid** |
| **8** | **H. u. Gregorio Marañón** | **Madrid** |
| **9** | **H. U. La Princesa** | **Madrid** |
| **10** | **H. del Mar** | **Barcelona** |
| **11** | **H. U. de Asturias** | **Oviedo** |
| **12** | **H. U. Marqués de Valdecilla** | **Santander** |
| **13** | **H. Clínico U. de Valladolid** | **Valladolid** |
| **14** | **H. U. Salamanca** | **Salamanca** |
| **15** | **Complejo H. de Navarra** | **Pamplona** |
| **16** | **H.U. HM Sanchinarro** | **Madrid** |
| **17** | **Complejo hospitalario de Toledo** | **Toledo** |
| **18** | **Clínica Universidad de Navarra (CUN)** | **Pamplona** |
| **19** | **H. General de Albacete** | **Albacete** |
| **20** | **H. General U. de Ciudad Real** | **Ciudad Real** |
| **21** | **H. U. Miguel Servet** | **Zaragoza** |
| **22** | **Complejo Asistencial U. de León** | **León** |
| **23** | **Hospital General U. de Valencia** | **Valencia** |
| **24** | **H. U. Arnau de Vilanova** | **Lleida** |
| **25** | **H Sant Joan de Deu de Manresa. Fundación Althaia** | **Manresa** |
| **26** | **H. U. Sant Joan de Reus** | **Reus** |
| **27** | **H San Pedro** | **Logroño** |
| **28** | **H. Dr Josep Trueta** | **Girona** |
| **29** | **H. Dr. Negrín** | **Las Palmas** |
| **30** | **H. U. Mútua Terrassa** | **Terrasa** |
| **31** | **H. U. Quironsalud Pozuelo** | **Madrid** |
| **32** | **H. U. Donostia** | **Donostia** |
| **33** | **H.U. Son Espases** | **Palma** |
| **Transfusion Centers** | | |
| **#** | **name** | **CITY** |
| **1** | **Centro de Transfusión de la Comunidad de Madrid (CTCM)** | **Madrid** |
| **2** | **Centro de Transfusión de las Fuerzas Armadas (CTFA)** | **Madrid** |
| **3** | **Banco de Sangre y Tejidos Aragón** | **Zaragoza** |
| **4** | **Banco de Sangre y Tejidos de Cantabria** | **Santander** |
| **5** | **Centro Comunitario de Sangre y Tejidos de Asturias** | **Oviedo** |
| **6** | **Banc de Sang i Teixits de Catalunya** | **Barcelona** |
| **7** | **Banco de Sangre de La Rioja** | **Logroño** |
| **8** | **Centro de Hemoterapia y Hemodonación de Castilla y León** | **Valladolid** |
| **9** | **Banco de Sangre y Tejidos de Navarra** | **Pamplona** |
| **10** | **Centro de Transfusión de Albacete y Cuenca** | **Albacete** |
| **11** | **Centro regional de Transfusión Toledo-Guadalajara** | **Toledo** |
| **12** | **Centro de Transfusión de Ciudad Real** | **Ciudad Real** |
| **13** | **Instituto Canario de Hemodonación** | **Las Palmas** |
| **14** | **Centro de Transfusión de Valencia** | **Valencia** |
| **15** | **Banc de Sang i Teixits de les Illes Balears** | **Palma** |
