# Supplementary material for: Evaluation of convalescent plasma versus standard of care for the treatment of COVID-19 in hospitalized patients: study protocol for a phase 2 randomized, open-label, controlled, multicenter trial
Source: Trials. 2021 Jan 20;22:70. doi: 10.1186/s13063-020-05011-9 (PMC7816149; doi:10.1186/s13063-020-05011-9)
Supplement: Supplementary file 2 — Additional file 2. Eleven Point Ordinal Scale. [file 13063_2020_5011_MOESM2_ESM.pdf]

**Appendix 2: Eleven Point Ordinal Scale**

0. Uninfected; no viral RNA detected.

1. Asymptomatic; viral RNA detected, limitation on activities.

2. Symptomatic; independent

3. Symptomatic; assistance needed

4. Hospitalized, no oxygen therapy.

5. Hospitalized, oxygen by mask or nasal prongs6. Oxygen by mask or nasal prongs

7. Intubation and mechanical ventilation, $pO_2/FiO_2 \geq 150$ or $SpO_2/FiO_2 \geq 200$

8. Mechanical ventilation $pO_2/FIO_2 < 150$ ($SpO_2/FiO_2 < 200$) or vasopressors

9. Mechanical ventilation $pO_2/FiO_2 < 150$ and vasopressors, dialysis, or ECMO

10. Dead
